# Gingival Bleeding of a High-Flow Mandibular Arteriovenous Malformation in a Child with 8-Year Follow-Up

**DOI:** 10.1155/2015/745718

**Published:** 2015-03-17

**Authors:** Elvira Ferrés-Amat, Jordi Prats-Armengol, Isabel Maura-Solivellas, Eduard Ferrés-Amat, Javier Mareque-Bueno, Eduard Ferrés-Padró

**Affiliations:** ^1^Service of Oral and Maxillofacial Surgery, Fundació Hospital de Nens de Barcelona, Consell de Cent 437, 08009 Barcelona, Spain; ^2^Department of Oral and Maxillofacial Surgery, Faculty of Dentistry, Universitat Internacional de Catalunya, Josep Trueta s/n, Sant Cugat del Vallès, 08195 Barcelona, Spain; ^3^Service of Pediatric Dentistry, Fundació Hospital de Nens de Barcelona, Consell de Cent 437, 08009 Barcelona, Spain

## Abstract

Intraosseous arteriovenous malformations (AVMs) in the head and neck region are uncommon. There are several types and they can have a wide range of clinical presentations. Depending on the blood flow through the AVM, the treatment may be challenging for the attending team and may lead to life-threatening hemorrhages. A clinical case report is presented. A 9-year-old girl, seen for gingival bleeding during oral hygiene, was found to have a high-flow AVM located within and around the mandible. Two-stage treatment consisted of intra-arterial embolization followed by intraoral injection of a sclerosing agent 8 weeks later. At the 8-year follow-up, imaging study showed no evidence of recurrent lesion inside or outside the bone. The final outcome is a correct occlusion with a symmetric facial result. This case shows that conservative treatment may be the first treatment option mostly in children. Arteriography and transcortical injection were enough to control the AVM.

## 1. Introduction

The classification of benign vascular lesions and the related terminology was confusing until Mulliken and Glowacki [1] differentiated this generic term into two entities: vascular malformations and hemangiomas (tumors). Hemangiomas are one of the most common soft tissue tumors in children [2], whereas vascular malformations occur much less frequently. The currently accepted classification was published by Mulliken and Glowacki in 1982 [1], later modified by Mulliken et al. [3], and accepted in 2014 by the International Society for the Study of Vascular Anomalies (ISSVA) http://www.issva.org/. Vascular malformations are related to an abnormality in embryonic development and are composed of ectatic vessels (venous, arteriovenous, or lymphatic vessels). They are classified according to the vessel affected and the amount of flow through the vessel: high flow (fistulas, arteriovenous malformations, and mixed malformations) and low flow (capillary, venous, lymphatic, and mixed malformations). Vascular malformations are present from birth, but they may be asymptomatic and go undetected at that time. Most of these lesions appear in the head, neck, and trunk regions [2], and they mainly affect the skin and scalp.

Of all vascular anomalies, AVMs are the most dangerous because they can be associated with life-threatening complications. Despite their benign histology, deep lesions can produce serious systemic signs and symptoms due to extensive arteriovenous shunting and soft tissue hypertrophy. However, AVMs usually produce more subtle signs as they grow. Mandibular AVM shows a wide variety of signs and symptoms, such as dental mobility, otalgia, secondary pain due to thrombosis, facial asymmetry, and cosmetic distress [4–6]. AVMs were classified in 1990 by the International Workshop for the Study of Vascular Anomalies as follows: stage I: cutaneous blush or warmth; stage II: bruit, audible pulsations, expanding lesion; stage III: pain, ulceration, bleeding, and infection; and stage IV: heart failure [7].

In this paper, we report a case of AVM of the mandible manifesting in the pediatric age, with a description of the clinical and imaging findings leading to the diagnosis and the strategy used for treatment.

## 2. Case Presentation

**Figure 1:**
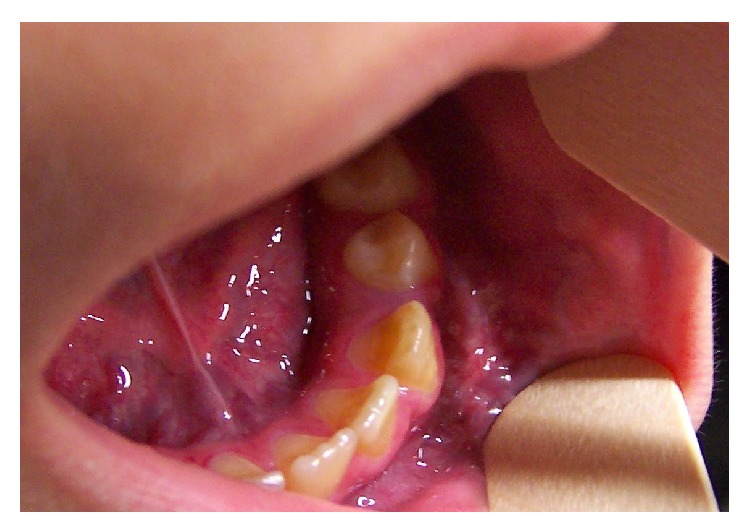
Initial intraoral picture.

A 9-year-old girl was received for consultation at the Maxillofacial Unit of Hospital de Nens de Barcelona. She had been referred because of bleeding around the permanent mandibular left first molar. The child had no relevant medical history. Her mother reported gingival bleeding in the left mandible for two months. The patient presented facial asymmetry, with increased size of the lower facial third on the left side. Intraoral examination showed gingival bleeding and an ecchymotic area extending from the canine to the retromolar area (Figure 1). On palpation, the vestibular gingiva soft tissue was found to be hot and pulsatile. The volume of mandibular bone was increased compared to the right side. Furthermore, the permanent mandibular left first molar was mobile. Panoramic radiography showed a poorly delimited, multiloculated radiolucency resembling soap bubbles, displacing the permanent mandibular left second molar and expanding the mandibular cortical bone, without destruction of the dental structures. The patient was referred to Vall d'Hebrón Hospital for a complete imaging study with computed tomography (CT) and magnetic resonance angiography (MRA).

**Figure 2:**
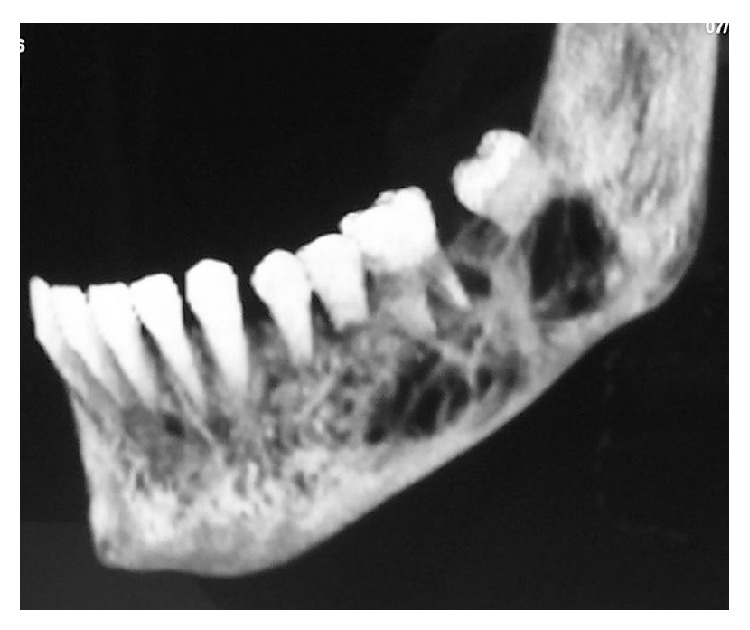
Preoperative CT scan.

**Figure 3:**
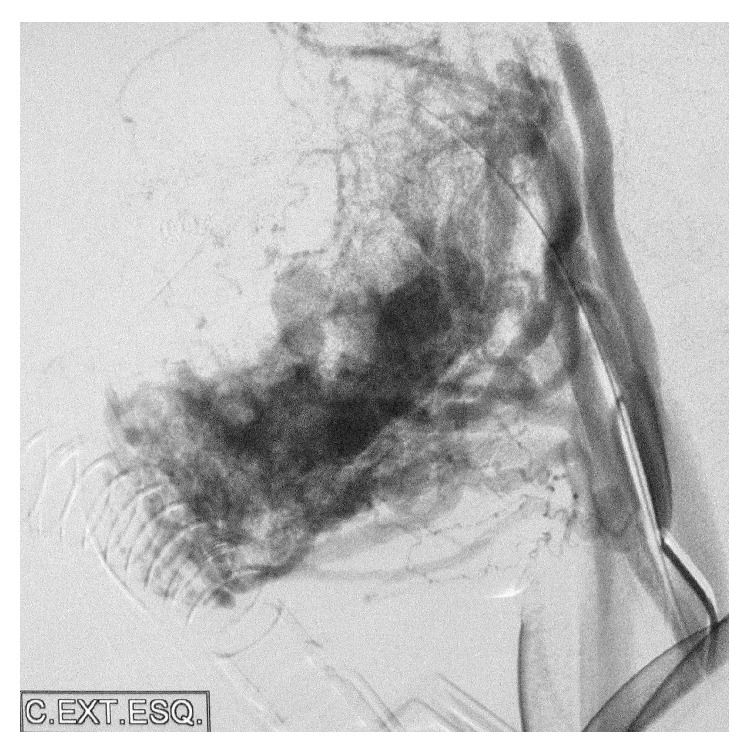
Preoperative angiography: left common carotid angiogram, lateral projection, and arterial phase showing extensive mandibular AVM with arterial supply from multiple sources and drainage into the dilated inferior alveolar vein.

CT confirmed the multiloculated appearance, bone expansion, and preservation of the dental structures within the affected area (from the canine to the left mandibular angle, involving the entire horizontal branch of the mandible) (Figure 2). Gadolinium-enhanced MRA confirmed the diagnosis of a high-flow intra- and perimandibular AVM (Figure 3). Analytical determinations in blood were normal. The findings obtained with these examinations established the diagnosis of a high-flow CAVM (capillary arteriovenous malformation), affecting the left lower third of the face, involving the body and branch of the mandible and the masticatory muscles, mucosa, and skin.

Interventional angiography through a femoral approach was decided as a diagnostic and therapeutic option. Angiography showed a high-flow vascular malformation with branches emerging from the facial artery, the lingual artery, and the inferior alveolar nerve artery. During the procedure, several branches were sclerosed with a 40 : 60 mixture of cyanoacrylate tissue adhesive (Glubran; GEM S.r.l., Viareggio, Italy) + Lipiodol.

**Figure 4:**
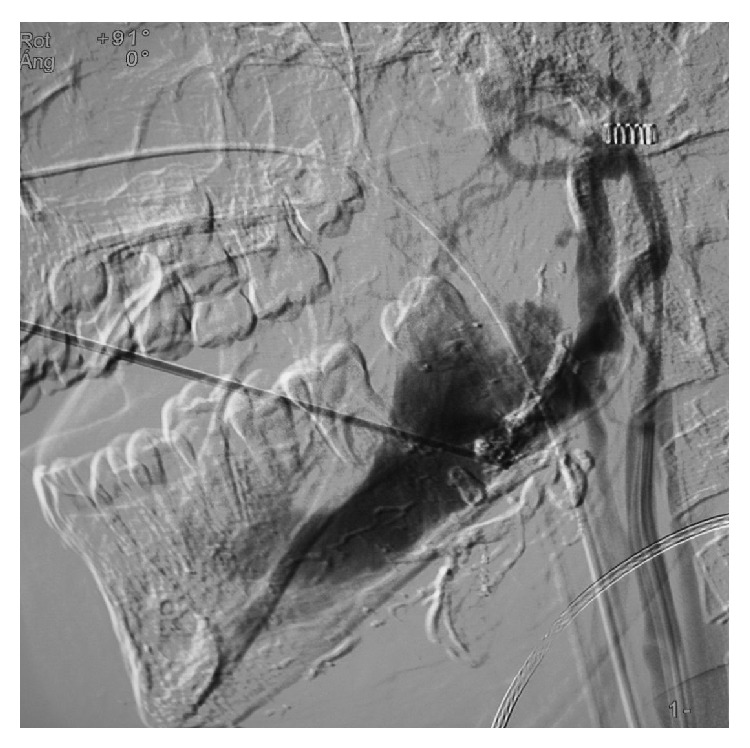
Intraoperative transcortical injection.

As only an angiographic embolization was not enough to solve the problem, a second embolization procedure with femoral access was scheduled 8 weeks later. This time in combination with an intraoral approach, transcortical puncture was used to access the interior of the mandible with a trocar under radioscopic control and angiographic support (Figure 4). An alcoholic solution of zein (Ethibloc; Ethicon, Johnson & Johnson, Switzerland) as sclerosing agent was then injected inside the body and the branch of the mandible, avoiding the tooth buds and roots, and the inferior alveolar nerve. Filling of the vascular lacuna was achieved.

**Figure 5:**
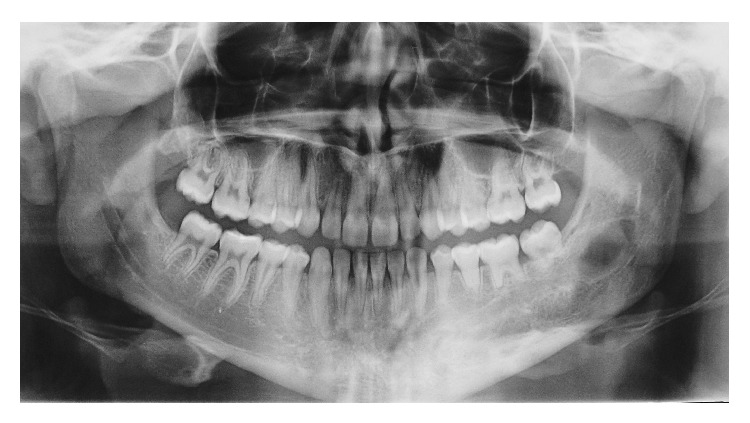
8-year follow-up X-ray.

**Figure 6 fig6:**
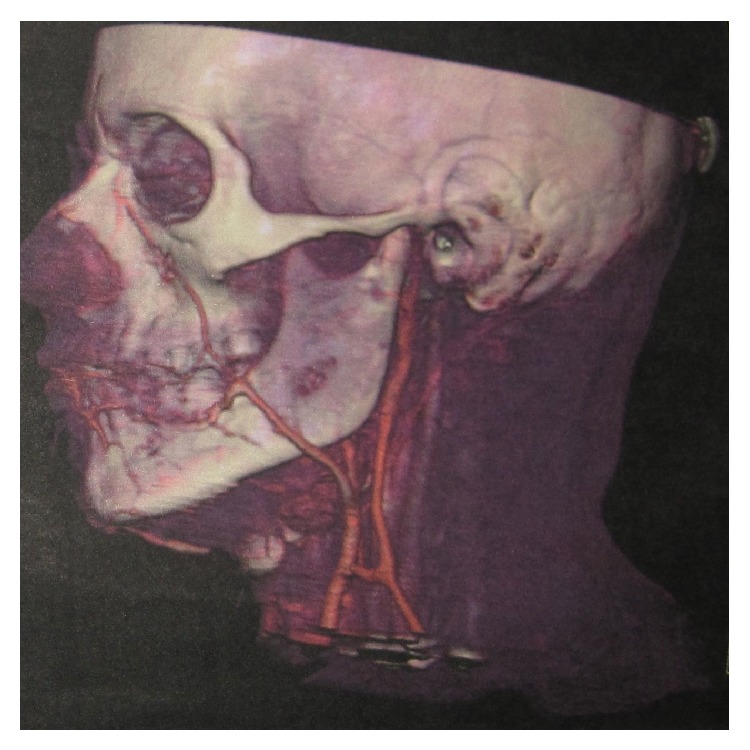
8-year follow-up angio-CT.

**Figure 7:**
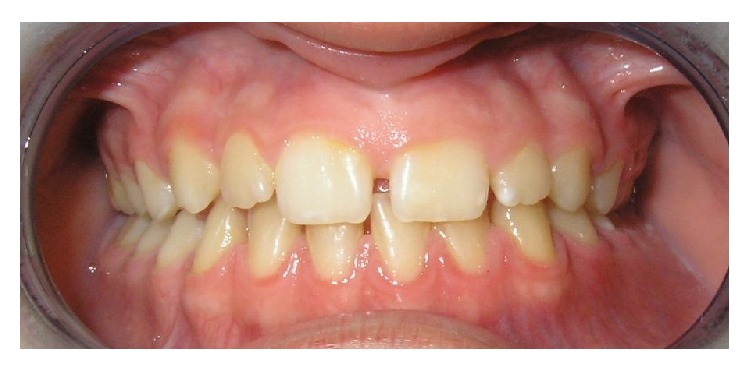
8-year follow-up intraoral picture.

Postoperative imaging showed control of the lesions with ablation of all branches. Follow-up consisted of physical examination, panoramic radiography, and MRI and CT studies. There has been no evidence of relapse to date, at 8 years of follow-up. The patient has functional occlusion with no deviation of the mandible or the occlusal plane. There are no aesthetic sequelae, and her permanent mandibular left second molar has erupted correctly; although radiographically they show some root resorption they have no significant mobility (Figures 5 [Fig fig6]–7).

## 3. Discussion

A high-flow AVM involving the mandible and surrounding soft tissue is extremely rare. The literature contains few cases and they are quite diverse; hence, the overall approach to take and therapeutic algorithm to follow in these patients remain uncertain. As reported by Corsten et al. [8], mandibular AVMs are unusual lesions that many dentists, oral and maxillofacial surgeons, head and neck surgeons, general practitioners, pediatricians, pediatric surgeons, plastic surgeons, ENT, and radiologists may not have encountered previously.

Suspicion of a maxillary or mandibular AVM should arise when the first clinical signs appear, including facial asymmetry, pain, swelling, local heat, tooth mobility, bluish discoloration of the mucosa and gums, or gingival or floor mouth bleeding in a patient with no history of predisposing factors, such as trauma or a known diagnosis of blood dyscrasia or oropharyngeal neoplasm [9]; Lamberg et al. [10] reported that intraosseous AVMs of the maxillofacial region are often diagnosed as a result of dental extraction or exfoliation, which produces torrential and life-threatening hemorrhage because the patient and physician are unaware of the lesion [11]. It is important to alert the medical and dental community about these lesions; although they are rare, a simple radiographic study combined with clinical examination may suffice to prevent fatal consequences. Any surgical manipulation except emergency procedures to control bleeding should be postponed until the patient is first stabilized and the vascular lesion is occluded by embolization. Biopsies and dental procedures are totally contraindicated due to the risk of fatal bleeding.

The diagnosis is usually made in conjunction with clinical and radiographic investigations. The presence of a mandibular radiolucency on panoramic radiography may suggest AVM as a differential diagnosis. CT angiography shows the lesion and the feeding vessels. MRI can additionally help to differentiate between tumors and malformations. Nonetheless, angiography is the reference standard diagnostic method, although it is more invasive [9]. Regarding the imaging, MR angiography is also effective in demonstrating the vascular supply to the lesion, avoiding the use of radiation in a child. Diagnostic angiography is usually performed at the same procedure as the therapeutic embolization.

High-flow lesions are a challenge for the attending team of professionals. The standard treatment for AVM has been endovascular embolization with subsequent surgical removal of the lesion [12], but nowadays several additional treatments have been described: superselective intra-arterial embolization (SIAE), sclerotherapy, radiotherapy, bone wax packing of bone cavities and curettage, surgical resection, or combinations of these therapies [9, 11, 13–21]. Some authors have used less aggressive techniques than surgery. For example, Liu et al. [22] reported on 8 cases of central AVM of the jaw treated with direct intraosseous glue sclerotherapy. The authors considered the technique to be safe and simple and described complete devascularization and reossification after single or multiple histoacryl injections. Bergeron et al. [23] also found combined endovascular and transcutaneous angioembolization with histoacryl to be effective for mandibular AVM treatment. Mandibular AVMs are usually arterial low venous malformations with single outflow vein physiology. That is the reason why they respond so well to direct transosseous embolization of the draining vein. This effectively closes all of the arteriovenous shunts. It has a very high rate of success and permanency. However, Motamedi et al. [15] suggested that surgical treatment is needed in addition to embolization. Unfortunately, preoperative embolization does not decrease the size of the resection [16]. Segmental resection has been recommended for the treatment of extensive bone lesions. Bone reconstruction has been suggested after AVM resection in the jaws to maintain teeth and temporomandibular joint function and offer biological bases for implants and prostheses [11]. Block resection has also been used in AVMs involving the floor of the mouth or the parotid gland, lip, and cheek to avoid an obvious effect on appearance. Thus, there is considerable evidence that surgical resection is an effective but also aggressive method for the treatment of AVMs.

Recently, minimally invasive surgical management proposals have emerged, such as cleaning the cavity through the alveolar process reported by Azzolini et al. [17], the buccal window approach proposed by Rattan and Sethi [18], and the cortical holes strategy described by Brusati et al. [19]. Wang and Huang [20] proposed curettage via the intraoral approach when the lesion is confined to the bone, which is particularly useful in hospitals with limited equipment, where advanced techniques and instruments are not available. The study of Chen et al. [13] describes various treatments for AVM in the oral and maxillofacial region in 28 patients. The authors concluded that bone wax packing of the bone cavity and curettage is a simple, safe, and effective method for the treatment of AVMs of the jaws. However, radiotherapy and sclerotherapy may not be effective methods for AVMs involving the soft tissue. In 2011, Gluncic et al. [21] reported a case of mandibular AVM treated by molar extraction and direct hydroxyapatite cement infusion into the mandibular cavity, which produced complete hemostasis and AVM obliteration. Surgical ligation, at the origin of both external carotid arteries, usually precludes any further endovascular access to these malformations and should be done only if it is considered the only feasible life-saving procedure, in an emergency situation, in a hospital where endovascular techniques are not available and the patient is not stable enough to be transferred to a larger facility [24–29].

The case presented of a young patient with a mandibular mass consistent with a large and complex AVM was successfully resolved with two sclerotherapy approaches: intra-arterial and a transcortical approach. Clinicians must be mindful not to consider cessation of hemorrhage as an indicator of complete cure, because vascular reconstitution can lead to regrowth of the AVM and subsequent hemorrhage. Careful follow-up of patients treated is needed before the procedure's long-term success can be assessed.

## 4. Conclusions

It is important to be aware of AVMs of the mandible so that the medical community and particularly dentists and pediatricians will take them into account in the differential diagnosis. The diagnosis is easily achieved with physical examination and imaging studies. Patients are usually diagnosed when the first clinical signs appear: pain, swelling, gingival bleeding, local heat, tooth mobility, or bluish discoloration of the mucosa and gums. A correct diagnosis is essential because treatment depends on it, and the proper choice of treatment is also a key factor. Patients with extensive or complex vascular anomalies should be treated by a multidisciplinary team that can provide appropriate, up-to-date treatment. Grouping of cases will facilitate clinical research in this line, with the aim of improving the therapy for this rare condition.
